# Ultra-Hypofractionated vs. Moderate Fractionated Whole Breast Three Dimensional Conformal Radiotherapy during the COVID-19 Pandemic

**DOI:** 10.3390/medicina58060745

**Published:** 2022-05-30

**Authors:** Olivera Ivanov, Aleksandra Milovančev, Borislava Petrović, Nataša Prvulović Bunović, Jelena Ličina, Marko Bojović, Ivan Koprivica, Milijana Rakin, Milana Marjanović, Dejan Ivanov, Nensi Lalić

**Affiliations:** 1 Faculty of Medicine, University of Novi Sad, 21000 Novi Sad, Serbia; olivera.ivanov@mf.uns.ac.rs (O.I.); natasa.prvulovic@mf.uns.ac.rs (N.P.B.); jelena.licina@mf.uns.ac.rs (J.L.); marko.bojovic@mf.uns.ac.rs (M.B.); milijana.rakin@outlook.com (M.R.); dejan.ivanov@mf.uns.ac.rs (D.I.); nensi.lalic@mf.uns.ac.rs (N.L.); 2 Department for Radiation Oncology, Oncology Institute of Vojvodina, 21204 Sremska Kamenica, Serbia; borislava.petrovic@df.uns.ac.rs (B.P.); ivankoprivica@gmail.com (I.K.); milana.marjanovic92@gmail.com (M.M.); 3 Department for Cardiology, Institute of Cardiovascular Diseases of Vojvodina, 21204 Sremska Kamenica, Serbia; 4 Faculty of Sciences, University of Novi Sad, 21000 Novi Sad, Serbia; 5 Center for Diagnostic Imaging, Oncology Institute of Vojvodina, 21204 Sremska Kamenica, Serbia; 6 Department for Abdominal and Endocrine Surgery, Clinical Centre of Vojvodina, 21000 Novi Sad, Serbia; 7 Clinic for Pulmonary Oncology, Institute for Pulmonary Diseases of Vojvodina, 21204 Sremska Kamenica, Serbia

**Keywords:** radiotherapy, ultra-hypofractionated radiotherapy, breast cancer, COVID-19

## Abstract

*Background and Objectives*: Reducing time of treatment during COVID-19 outbreaks has been recommended by the leading Radiation Oncology societies. Still minimizing radiation induced tissue toxicity is one of the most important issues in breast cancer patients. The study aimed to investigate compliance, clinical and dosimetry normal tissue toxicity, and cosmetic results between moderated and ultra-fractionated regimes for breast cancer patients during COVID-19 pandemic. *Materials and Methods*: This pilot prospective randomized study included 60 patients with early breast cancer after preserving surgery, 27 patients advocated to ultra-hypofractionated whole-breast three dimensional (3D) conformal radiotherapy of 26 Gy in 5 fractions over 1 week and 33 patients with moderate fractionated breast 3D conformal radiotherapy patients between March 2020 and July 2020, during the COVID pandemic outbreak. The compliance to treatment, dosimetric parameters, acute and late skin toxicity, subcutaneous tissue toxicity, cosmetic results and clinical follow up for 18 months for the two regimes were analyzed and compared. *Results:* When two regimes were compared 5 fraction group had significantly lower prevalence of newly infected cases of SARS-CoV-2 and thus delayed/interrupted treatment (*p* = 0.05), comparable grade 1 CTCAE v5, acute skin toxicity (*p* = 0.18), Grade 1 Radiation Morbidity Scoring Scheme (RESS) subcutaneous tissue toxicity (*p* = 0.18), Grade 1 RESS late skin toxicity (*p* = 0.88) and cosmetic results (*p* = 0.46). Dosimetric results reveled that patients in 5 fraction group received significantly lower median ipsilateral lung doses (*p* < 0.01) in addition to left breast cancer patients that received significantly lower median heart dose (*p* < 0.01) and median left anterior descending artery (LAD) dose (*p* < 0.01). *Conclusion:* Ultra-hypofractionated radiotherapy for breast cancer is comparable to moderate hypofractionation regimen regarding grade 1 acute skin toxicity, grade 1 subcutaneous tissue toxicity, late skin toxicity and cosmetic results. Application of ultra-hypofractionated radiotherapy with significantly lower radiation doses for lung and heart could be crucial in reducing the risk of acute/late pulmonary and heart radiation-induced toxicity.

## 1. Introduction

From the begging of the Coronavirus disease 2019 (COVID-19) pandemic, the provision of healthcare services and their accessibility has been a major problem worldwide [1]. In order to avoid long-term complications, decrease morbidity and mortality, new regimes need to be adopted to facilitate prompt and timely access to treatment, especially in vulnerable categories of cancer patients. Different barriers such as financial, organizational, social can reduce access to treatment [2], all these reasons are for sure exaggerated in pandemic times. The COVID-19 pandemic faced the scientific community, and more particularly healthcare professionals with unprecedented challenges [3]. In a survey by the American Society for Radiation Oncology (ASTRO) [4] that included 222 radiation oncology leaders during early COVID outbreak, centers reported multiple challenges such as personal protective equipment and financial declines, but majority of clinics continued to treat patients without compromise following rapid implementation of protective safety measures and process adaptations that allow them to continue to provide high quality care to cancer patients. Radiation oncology teams adapted care plans to provide shorter treatment courses to reduce the number of visits to a health care facility, physicians delayed radiation therapy for some cancers, but although continued services, patient volume decreased substantially by 85% due mostly to treatment delays/deferrals for certain diseases, as well as fewer patients being referred for radiation therapy. Many practices experienced staff reductions due various reasons. In response to the outbreak, a workgroup convened by ASTRO leadership issued clinical guidance to help radiation oncology teams continue providing safe, high quality cancer care while also minimizing the risks of COVID-19 exposure for patients and clinical staff. In European SocieTy for Radiotherapy and Oncology (ESTRO) [5] survey European radiation oncology department heads reported similar results, with 60% decline in patient volume, telemedicine was used in 78% of the departments, and shortages of personal protective equipment in more than half of the departments.

Breast cancer is the leading cause of death from cancer in women worldwide [6]. Moderate hypofractionation for breast cancer radiotherapy has been established as a standard of care in many radiotherapy centers worldwide. Several meta-analyses and randomized trials have been published over the last ten years, all demonstrating that moderate hypofractionation modality radiotherapy regime (40–42.5 Gy in 15–16 fractions over 3 weeks) is a safe and effective in local control, toxic effects, and cosmetic outcomes after breast-conserving surgery and mastectomy compared to conventional fractionation over 5 weeks [7,8]. As medical advances are pushing radiotherapy via less fractions with higher dose per fraction, especially in this period of COVID 19 pandemic, it becomes very important to estimate this risk of tissue toxicity in order to provide good cosmetic results which are very important for patients’ quality of life. However, due to various reasons, ultra-hypofractionation for breast cancer external beam radiotherapy has been increasingly investigated during the last decade [9,10]. Different treatment schedules have been used, but mostly 26 Gy or 27 Gy in 5 fractions over 1 week. Both regimens were proved non-inferiority for local control and similar results for late tissue toxicity, but cosmetic results were reported as worse in 27 Gy given over 1 week when compared to moderate hypofractionation regimens. Currently, there are still limited data regarding skin, heart, and lung toxicity of 1-week regimes, and scares data about different center’s adoption in everyday clinical settings. Acute skin toxicity which is manifested as radiation dermatitis is one of the most common adverse events in breast cancer radiotherapy and yet there isn’t enough evidence whether patients who receive ultra-hypofractionation are at higher risk for acute or late skin toxicity [11].

Prevention or minimizing radiation induced heart and lung toxicity is one of the most important issues in breast cancer radiotherapy with the goal to reduce morbidity, bearing in mind that breast cancer patients are usually long-term cancer survivals [12,13]. In radiotherapy treatment planning, maximum and mean doses for organs at risk are important for predicting possibility of acute and late toxicity. Doses to heart and lungs are routinely measured and reported in 2 Gy per fraction biologically equivalent dose (BED) to ensure that exposure to these organs is below the tolerance limits. More precision in heart exposure estimation is by measuring the dose to left anterior descending artery (LAD,) particularly for left-sided breast cancer [14,15]. It remains unclear whether there is a higher normal tissue acute sensitivity to total dose rather than dose per fraction and is there a higher risk for late normal tissue toxicity using ultra-hypofractionated radiotherapy regimen. Hypofractionated, shorter regimens gain in pandemic settings, a new reason for being included in the general protocols of social distancing and limiting the patient’s visits in the radiotherapy department.

The study aimed to investigate and compare compliance, skin and subcutaneous tissue toxicity, cosmetic and dosimetry results between moderated and ultra-fractionated regimes for early breast cancer patients during COVID-19 pandemic.

## 2. Materials and Methods

Eligble female patients (N 76) were offered to participate in pilot prospective randomized clinical study in addition to regular treatment. Inclusion criteria were diagnosed early breast cancer (T1-3 N0-1 M0) requiring radiotherapy with previously preserving surgery and complete macroscopic resection of invasive carcinoma. Exclusion criteria were age under 40 years, planned sequential boost, postmastectomy irradiation, and indication for nodal treatment. The study was approved by Institutional Scientific and Ethical Board of tertiary care Oncology Institute of Vojvodina under the number 4/20/1-1139/2-2 and conducted according to the principles of Helsinki Declaration. Overall, 66 participants gave informed consent and randomized.

**Figure 1:**
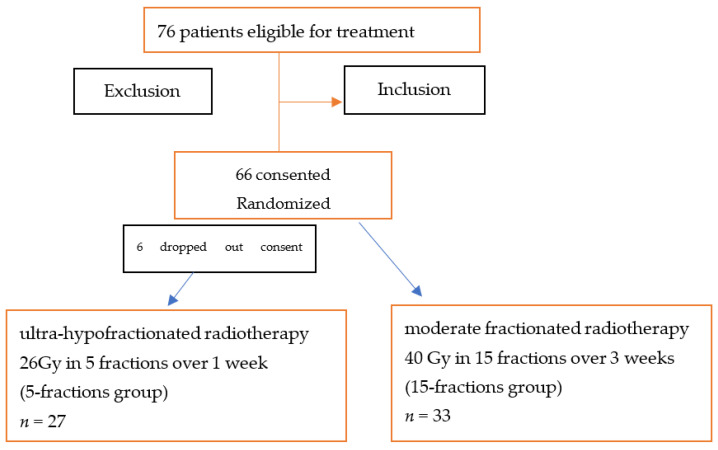
Study flow chart.

The patients were advocated to ultra-hypofractionated whole-breast three dimensional (3D) conformal radiotherapy of 26 Gy in 5 fractions over 1 week (5-fractions group) or moderate fractionated whole left-sided breast 3D conformal radiotherapy, (Figure 1, Flowchart.) between March 2020 and July 2020, during the COVID pandemic outbreak.

In the group allocated for ultra-hypofractionated whole-breast three-dimensional (3D) conformal radiotherapy of 26 Gy in 5 fractions over 1 week, 6 patients withdrawn consent, and finaly 27 patients were included. Among them, nine patients had left-sided breast cancer.

The 33 patients were advocated to moderate fractionated whole left-sided breast 3D conformal radiotherapy during the same timeframe. Patients in this group received 40 Gy in 15 fractions over 3 weeks (15-fractions group).

The Common Terminology Criteria for Adverse Events (CTCAE) version 5.0 was used to grade the severity of radiation-related skin, lung and heart toxicities [16]. Acute treatment toxicity was defined as any adverse effect that occurs within 6 months from the treatment beginning. Late toxicity was defined as any adverse effect that occurred after 6 months or more of treatment completion. Acute skin toxicity (radiation dermatits) was diagnosed by a radiation oncologist and treated according to the clinical protocol. Radiation Therapy Oncology Group/European Organization for Research and Treatment of Cancer (RTOG/EORTC) Late Radiation Morbidity Scoring Scheme (RESS) was used to grade late skin and subcutaneous tissue toxicity [17]. The cosmetic effect of the radiotherapy was measured by the patients and the radiation oncologist via four-point scale: 1-appearance of the breast didn’t change, 2- the appearance of the breast changed a little, 3- the appearance of the breast changed to a greater extent and 4- appearance of the breast changed very much.

All patients were examined clinically during the follow-up period, 2 weeks after the end of radiotherapy, and then after 2, 6, 12, and 18 months after treatment termination for sympthoms and signs of deterioration of previous skin, lung, or heart disease.

For all 60 patients, average and total time (minutes) that patients spent at Radiation Oncology Department (ROD) during treatment days were recorded for each patient. Antigenic or Real-Time Reverse Transcription-Polymerase Chain Reaction (RT-PCR) on SARS-CoV2 were used for testing. Before radiotherapy all patients were tested. If there were any suspicion of COVID-19 infection in the presence of typical symptoms during radiotherapy, the treatment was postponed until the confirmatory tests are done. If COVID-19 confirmed, infected patients, did not start radiotherapy, and discontinue the treatment that began before, for 14 days or two negative PCR tests.

All cases of COVID 19 disease occurrence during the treatment were recorded for both groups. The patients fulfilled the questionnaire at the end of the treatment. Questionare had questions about on personal opinion on the convenience of the treatment (easy, medium, hard), compliance (delayed/interrupted tretment, unmodified treatment).

### 2.1. Treatment Details

The treatment protocol was the same for the 5-fractions and 15-fractions group. Active breathing control was used for patients with left-sided breast cancer. Patients were scanned in supination with a breast immobilization device (Wing-board, Civco, Kalona, IA, USA). A spiral CT simulation was performed from the mandible angle to the 5 cm below the visible breast tissue with 2mm slice thickness. All the scanned images were uploaded to the treatment planning system (TPS) Eclipse and Aria, Varian Medical Systems INC, Palo Alto CA USA, or Monaco TPS ver.5.11.02, Elekta, Stocholm, Sweden. Target and organs at risk delineation were according to the ICRU 50 and 62 recommendations. Clinical target volume (CTV) included whole breast tissue and margin of 10 mm was added accounting for set-up error to create a planning target volume (PTV). Delineation of lungs, heart, LAD, skin and bone marrow was performed as organs at risk (OAR) constrains were V8 < 15% (ideal) and V8 < 17% (acceptable) for the ipsilateral lung, V1,5 Gy < 30%, and V7 < 5% for the heart [18]. Mean heart dose had to be less than 3 Gy. The organ at risk (OAR) constraints are based on FAST Forward trial (1 week regime) and START trials (3 week regime) [4,6,7]. Median doses (D mean) to the OAR and particular volumes were measured in both groups. For the ipsilateral lung, MLD, total volume expressed in cm^3^, V20 and V8 volumes were measured. Median dose, total heart volume and V8 were recorded for left-sided breast cancer patients’ subgroup of 5-fractions group and whole 15-fractions group. Median and maximal doses for the LAD were measured. Verification imaging was obtained for each fraction in 5-fractions group, using MV or kV X-rays. In 15-fractions group verification imaging was obtained according to the radiation oncologist preference, minimally for the first three fractions following once-weekly imaging.

### 2.2. Statistical Analysis

Descriptive statistics are presented as percentages, mean ± SD or median and interquartile range (IQR). Independent-Samples t-test was used to compare age and other continous variables between two groups. Chi-squared and Fisher-Freeman-Halton tests were used to identify differences for categorical variables between two groups where apropriate. Mann-Withney U test was used to compare doses to the lung, heart, and LAD between two groups. Shapiro Wilk test was used to test normality of distribution. *p*-value less than 0.05 was considered statistically significant. Statistical analysis was performed using SPSS 23.0 for Windows (IBM Co., Armonk, NY, USA) and Jamovi V2.2.2 computer statistical software. Retrieved from https://www.jamovi.org (accessed on 1 April 2022), Sydney, Australia.

## 3. Results

The study included 27 patients aged 47–81 years, mean 62.8 ± 8.6 in group treated with ultra-hypofractionated 5-fractions radiotherapy vs. 33 patients aged 45–83 years 63.6 ± 9.8, *p* = 0.7, treated with moderate hypofractionated 15-fractions radiotherapy. General patients’ characteristics are presented in Table 1.

There was no statistical significance in disease stage or comorbidities when groups were compared.

### 3.1. Compliance and Special Considerations of Ultra-Hypofractionated Regimen during COVID Pandemic

The average time that patients spent at ROD daily during a 5-fraction schedule in five treatment days was 30.3 ± 2.9 min vs. 29.7 ± 2.5 min in the 15-fractions group, *p* = 0.45. Average time that patients spent at ROD during full treatment was 156.1 ± 19.7 min in 5-fractions group compared to 463 min ± 64.6 in the 15-fractions group *p* < 0.01. When analyzing the questionnaire on personal opinion on the convenience of the treatment, 15 (55.6%) of the patients in the 5-fractions group experienced treatment as easy, compared to 8 (24.2%) patients in the 15-fractions group, *p* = 0.01. There was significant difference in compliance between groups due to new cases of patients infected with SARS-CoV-2. Treatment was delayed/interrupted in 2 (7.4%) of patients in 5-fractions group compared to 9 (27.2%) of patients in the 15-fractions group, *p* = 0.05. Four patients were found COVID-19 positive before treatment initiation and treatment was delayed in one patient (3.7%) in 5-fractions group vs. 3 (9%), *p* = 0.41 in 15-fraction group. The treatment discontinuation for confirmed COVID-19 during treatment had one patient (3.7%) for 14 days in 5-fractions group vs. 6 (18.2%), *p* = 0.08 in 15-fraction group. The mean treatment discontinuation was 17 ± 4.4 days in in 15-fraction group, 2 patients had COVID-19 related pneumonia, but eventually recovered, one in 21 days and other in 24 days.

### 3.2. Normal Tissue Effects

Acute skin toxicity was observed in 63.3% of cohort, with grade 1 being the most prevalent in 53.3% of cases. There were no significant difference between groups in the prevalence of grade 1, 62.9% vs. 45.5%, *p* = 0.18. Grade 2 was found in low prevalence of 10%, and it was more often observed in 5-fractions group 18.5% vs. 3%, *p* = 0.04. None of the patients developed acute skin toxicity grade ≥3. Data are presented in Table 2.

There were no significant differences between groups in prevalence of late skin toxicity 33.3% in 5-fractions vs. 18.2% in 15-fractions radiotherapy schedule, *p* = 0.18.

Grade 1 subcutaneous tissue toxicity (fibrosis and field contracture) was seen in 25% of cohort cases, without significant differences between group 25.9 vs. 24.2%, *p* = 0.88, RESS 2 was more frequent in 5-fractions group, 18.5% compared to 0% patients in 15-fractions group, *p* = 0.02. There was no significant difference in cosmetic results between two groups (*p* = 0.46) (Table 2).

### 3.3. Ipsilateral Lung

There was statistically significant difference in median dose to the ipsilateral lung 2.9 (IQR 1.4) in 5-fractions group vs. 4.8 Gy (IQR 2) in 15-fractions group, *p* < 0.01. When lung doses were compared 5-fractions group received significantly lower dose in V 20 4.8 (4.9) vs. 8.7 (6.3) %, *p* < 0.01 and V 8 10.6 (7) % vs. 14.5 (7.2) %, *p* < 0.01.

These results are shown in Table 3. None of the patients developed radiation pneumonitis or deterioration of previous lung disease in the follow up.

### 3.4. Heart and LAD

Median heart and LAD doses were compared among subgroup of patients with left-sided breast cancer alone in 5-fractions group and whole 15-fractions radiotherapy group (Table 3). Statistically significant difference was obtained in full received dose, median of MHD 0.9 (0.4) in 5-fractions group compared to 2.1 Gy (1.4) in 15-fractions group *p* < 0.01. The 5-fraction group received significantly lower V8 dose volume of 0.7% (1.3) compared to 4.1% (4.4) in 15-fractions group, *p* = 0.02 and lower median LAD dose 2.3 (1.9) vs. 10.1 (8.2), *p* < 0.01 and LAD max 10 (5.8) vs. 35.3 Gy (18.2), *p* < 0.01. None of the patients developed radiation induced heart disease. Finally, none of the patients experienced deterioration of previous heart disease.

## 4. Discussion

A longer duration of the treatment carries a higher risk of infection, in our study patients in 15 fraction group had significantly higher prevalence of newly infected cases with SARS-CoV-2 and thus delayed/interrupted treatment. Furthermore, they perceptive for treatment convenience was easier in this 5-fractions group, which is also very important as it can affect compliance to treatment. To our knowledge, this is the unique published study that compared duration of treatment and patient satisfaction among mentioned different radiotherapy schedules during early COVID-19 outbreak. The COVID 19 outbreak has significantly influenced all aspects of medical service, especially oncological departments and its patients who are prone to infection during the treatment. Infection with SARS-CoV-2 during treatment requires demission of the patient until recovery. Shortening or delay of radiotherapy treatment, safely, whenever possible, was the recommendation of the leading Radiation Oncology societies after the pandemic outbreak [18]. Radiation therapy for breast cancer usually lasts several weeks and brakes during the treatment are not desirable, in fact, pausing the treatment may influence oncological outcome of the patient.

Acute skin toxicity was highly prevalent in 63.3% of cohort. Grade 1 CTCAE v5 was seen in the majority of cases (53.3%), with one-week regime being comparable without statistically significant difference when compared to 3 weeks regime. Considerably lower prevalence of 10% grade 2 CTCAE v5 was observed in cohort, though we want to point out the significantly higher rate of Grade 2 skin toxicity (acute and subcutaneous) for the 5-fractions scheme. One week regime was also proved to be comparable without significant difference in term of late skin toxicity, Grade 1 subcutaneous tissue toxicity and cosmetic results although Grade 2 RESS subcutaneous tissue toxicity was more frequently seen in 5-fractions group (*p* = 0.02). It is documented that up to 95% of patients who undergo radiotherapy will develop moderate-to-severe skin reactions [19]. Randomized trials showed that 3-week schedule of 40 Gy had less acute and late skin toxicity events compared to the conventional 5-week schedule radiotherapy for breast cancer [20,21,22]. In FAST-Forward trial late normal tissue skin effect were worse for 27 Gy compared to 40 Gy at 5 years follow-up, but similar for 26 Gy in 5 fraction schedule [9].

In FAST-forward trial patients aged at least 18 years were eligible, however, they were ranged to 6 age groups. The proportion of women age < 50 was 14.6%, 13.9% and 15.8% for three study groups-40 Gy, 27 Gy and 26 Gy, respectively. Effectiveness of 26 Gy in 5 fractions regimen in younger ages should be evaluated in further prospective clinical trials.

Previously published studies also showed that higher dose per fraction was not associated with higher incidence of both early and late skin toxicity in breast cancer radiotherapy [19,23]. Factors as tumor boost, older age, tumor and breast size or diabetes mellitus could play an important role in both early and late radiation induced skin toxicity [24,25,26]. Different radiotherapy techniques such as intensity-modulated radiotherapy (IMRT), volumetric arc therapy (VMAT), accelerated partial breast irradiation, simultaneous integrated boost, and prone positioning could play an important role in radiation dermatitis development [11]. In this study, all of the patients were irradiated in supine position with 3D conformal radiation therapy, so there is limitation regarding the impact of different radiotherapy techniques. Acceptable toxicity was obtained by classical 3D conformal technique.

Radiation-induced changes in normal tissues are triggered with an acute inflammatory response with release of cytokines, tumor necrosis factor (TNF), growth factors, etc., which causes vascular endothelial damage and infection –like symptoms at first. Later, activation of the myofibroblasts cause tissue fibrosis and may result in myocardial or lung fibrosis or stricture of the blood vessels. Radiation pneumonitis, lung fibrosis, ischemic heart disease or valvular disease are the most significant complications of breast radiotherapy that can impair patients ‘quality of life usually after the treatment’ [13]. In this study, symptomatic radiation pneumonitis, fibrosis or other radiation-induced lung symptoms were not recorded during follow-up period. Mean doses to the ipsilateral lung, dose volume V8 and dose volume V20 were significantly lower in 5-fractions group, regardless of the fact that lung volumes received higher dose per fraction compared to 15-fractions group. In a large study that included 1847 women who received adjuvant RT for breast cancer it was concluded that hypofractionated schedule significantly reduce the ipsilateral lung dose compared to standard fractionation [27]. We assumed that this is due to the lower total dose and overall treatment time that allows normal tissue to repair, which is from that point of view encouraging for implementation of ultra-hypofractionated schedule. It is possible that patients who undergo 5-fractions radiotherapy schedule could have even less incidence of pneumonitis and lung fibrosis that correlates with significantly lower doses in these patients.

In our study 5-fractions group received significantly lower doses in mean heart dose, mean or maximal LAD dose. Symptomatic radiation-induced heart disease was not recorded in follow-up period of 18 months. Exposure of the heart to radiation during radiotherapy increases the subsequent rate of ischemic heart disease for breast cancer patients [28]. It is expected that left-sided breast cancer patients could experience radiation-induced heart toxicity [29]. Henson et al. found that relative increase in cardiac mortality from cardiac exposure during breast cancer radiotherapy was higher in younger women, lasted into the third decade after exposure and was higher when chemotherapy was also given [30]. Special radiotherapy techniques such as deep-inspiration breath hold, active breathing control via specialized device or respiratory gating are necessary to decrease dose/volume ratio of the heart. Recent study documented that the cumulative incidence of coronary events increased by 16.5% per Gy of mean heart dose and that the volume of left ventricle receiving 5 Gy was predictor of acute coronary events [31]. In our study, all of the patients fulfilled the criteria that 30% of the heart volume received less than 1.5 Gy. On the other hand, it is doubted whether doses higher than 2 Gy per fraction can trigger inflammatory reaction that promotes vessel atherosclerosis and consequently cause more late adverse effects of the radiotherapy. Results of previous studies confirmed that radiation dose distribution to cardiac subvolumes such as LAD and left ventricle better correlate with cardiac toxicity compared to mean heart dose [32]. Jacob et al. consider the distribution of doses within cardiac substructures (left ventricle and LAD) rather than just the MHD [33]. Since there are no established guidelines for LAD dose constraints, especially not for different regimes, the local clinical protocol follows LAD dose constraints as recommended by Danish breast cancer group- HYPO trial, advocating for LAD max < 20 Gy in 50/25 Gy regime [34]. Significant decrease in mean and maximal LAD dose in 5-fractions group of our study could implicates that this group of patients will experience less cardiac toxicity compared to patients who receive moderate hypofractionation. It is noteworthy that synergistic effect of systemic treatments of breast cancer and radiotherapy should be taken into account for cardiotoxicity. Further evidence is awaited from long –term follow-up of the patients who receive 26 Gy in 5 fractions schedule regarding radiation-induced heart disease.

It is obvious that total doses to the organs at risk are lower in 5-fractions schedule, but it is very important to determine the exact dose to each organ at risk volume/subvolume in order to optimize RT plan and to lower possible toxicity of the treatment. Radiation induced heart or lung disease usually manifests several years after treatment, so an annual check-up is recommended for patients who had cardiovascular comorbidities before radiotherapy as well as for the patients who develops symptoms during and in the first 6 months after end of the therapy. Long-term monitoring is needed for patients with respiratory symptoms that occur during or after radiotherapy with special attention to the patients with previous chronic obstructive lung disease or other lung disease.

FAST and forward was the first high quality randomized clinical trial on 4100 patients that recommended 5 days schedule to be used in the majority of early breast cancer patients. FAST and forward is randomized clinical trial testing a 1-week course of curative whole breast radiotherapy against a standard 3 week schedule in terms of local cancer control and late adverse effects in patients with early breast cancer. Overall, the Fast-Forward findings suggest that 1week course produces similar results to the standard 3-week treatment, both in terms of local control and of side effects. After high quality randomized trial, other randomized and observational studies are needed in the meanwhile to confirm/deny results of the study that launched the treatment. The 5-regime schedule is still not widely adopted as a regular treatment in Radiotherapy centers, this schedule has encountered resistance and concern in clinical implementation among radiotherapy centers worldwide, mainly due to short follow up of 5 years. The results for 10 years follow up are still waiting. Reported results in our study are comparable and confirm results of FAST and forward study with adding additional value of dosimetry parameters comparison between groups. This treatment especially gained in value in time of COVID-19 pandemic when shorter treatment schedules are recommended in order to avoid patient and staff COVID-19 infection. In the time of COVID-19 pandemic, any additional data of modified treatments outcomes are valuable.

There are several limitations of the current study. First, small sample size, especially for the left-sided breast cancer group and short follow-up period for late toxicity weakens the conclusion of the study. Finally, our study was done at single-center so further studies with longer follow-up are needed to involve more institutions to eliminate potential bias. The study is approved by Institutional board and randomized but not registered in clinical trials.

## 5. Conclusions

We postulate that ultra- hypofractionated radiotherapy can be a choice of treatment in time of COVID-19 and maybe in case of other pandemic outbreaks as it provides better compliance to treatment in patients with early breast cancer. The current study demonstrates that ultra-hypofractionated radiotherapy for breast cancer is comparable to moderate hypofractionation regimen regarding grade 1 acute skin toxicity, grade 1 subcutaneous tissue toxicity, late skin toxicity and cosmetic results. Application of ultra-hypofractionated radiotherapy with significantly lower radiation doses for lung and heart could be crucial in reducing the risk of late pulmonary and heart radiation-induced toxicity. Studies with more patients included and longer follow-up are needed to verify these early findings.

## Figures and Tables

**Table 1:** Age, stage and comorbidity distribution in two observed groups.

Parameter		5-Fractions Group *n* = 27	15-Fractions Group *n* = 33	*p*-Value
**Age (years)**		mean 62.8 ± 8.6 (47–81)	mean 63.6 ± 9.8 (45–83)	0.75
Stage *n* (%)	Stage I	11 (40.7%)	13 (39.4%)	0.91
	Stage II	16 (59.3%)	20 (60.6%)	
Hystory of Comorbidities *n* (%)	Lung	3 (11.1%) *	4 (12.1%) *	0.90 * 0.67 **
	Heart	10 (37%) **	14 (42.4%) **	

Legend: comparison of subgroups *p* value: * related to Lung diseases, ** related to Heart diseases.

**Table 2:** Normal tissue effects between groups.

	Gradus	5-Fractions Group *n* = 27	15-Fractions Group *n* = 33	*p*-Value
Acute skin toxicity (CTCAE)	1 CTCAE v5	17 (63%) *	15 (45.5%) *	0.18 * 0.04 **
	2 CTCAE v5	5 (18.5%) **	1 (3%) **	
Late skin toxicity (RESS-RTOG/EORTC)	1 RESS	9 (33.3%)	6 (18.2%)	0.18
Subcutaneous tissue toxicity (RESS-RTOG/EORTC)	1 RESS	7 (25.9%) *	8 (24.2%) *	0.88 * 0.01 **
	2 RESS	5 (18.5%) **	0 (0%) **	
Cosmetic results scale	1	14 (51.9%)	22 (66.7%)	0.46 ^c^
	2	4 (14.8%)	5 (15.2%)	
	2	9 (33.3%)	6 (18.2%)	

CTCAE-Common Terminology Criteria for Adverse Events. RESS-RTOG/EORTC Scoring Schema. ^c^—Fisher-Freeman-Halton test. One * or ** mark wich values were compared and p value related to * or **.

**Table 3:** Comparison of dosimetric parameters between groups.

		*n*	5-Fractions Group				15-Fractions Group				*p*-Value
			*n*	Min	Max	Median (IQR)	*n*	Min	Max	Median (IQR)	
**Ipsilateral lung dose**	Median dose (Gy)	**60**	27	0.7	4.9	2.9 (1.4) 4.8 (EQD2)	33	1.2	8.9	4.8 (2) 5.4 (EQD2)	<0.01 ^d^
	V20 (%)			0	12.6	4.8 (4.9) 7.9 (EQD2)		0.3	19.8	8.7 (6.3) 9.7(EQD2)	<0.01 ^d^
	V8 (%)			0.5	20.3	10.6 (7) 17.3(EQD2)		1.7	27.6	14.5 (7.2) 16.4(EQD2)	<0.01 ^d^
**Subgroup with only left breast cancer**											
**Heart dose**	Median dose (Gy)	42	9	0.6	1.3	0.9 (0.4) 1.4(EQD2)	33	0.7	5.5	2.1 (1.4) 2.4(EQD2)	<0.01 ^d^
	V8 (%)			0.1	2.9	0.7 (1.3) 1.1(EQD2)		0	14.6	4.1 (4.4) 4.6(EQD2)	0.02 ^d^
**LAD dose**	Median dose (Gy)			0.8	10.1	2.3 (1.9) 3.8(EQD2)		1.9	24.8	10.1 (8.2) 11.4(EQD2)	<0.01 ^d^
	Max dose (Gy)			3.3	23.2	10 (5.8) 16.4(EQD2)		7.1	40.7	35.3(18.2) 40(EQD2)	<0.01 ^d^

^d^—Mann Whitney U test; IQR-Interquartile Range, EQD2- equivalent dose in 2 Gy fractions (EQD_2_), LAD-left anterior descending artery.

## Data Availability

Data are available upon reasonable request to corresponding author.
